# The Hospital World

**Published:** 1911-05-27

**Authors:** 


### The Hospital World.

### Gift to an Old Dispensary.

Miss Priscilla Garratt Morton, of Horbury, near Wakefield, has left £100 to the Pimlico Royal Dispensary, London, in memory of her late father, Edward Morton, M.D., the founder and first physician of the dispensary, which began its existence in 1831. Before it moved to Eccleston Street we understand that it had its quarters in Buckingham Palace Road.

### Surgeon's Resignation at Salford.

Mr. E. T. Milner, Honorary Surgeon to the Salford Royal Hospital, has been compelled to resign his appointment on the score of "continued ill-health." At the monthly meeting of the Board, over which Mr. Lawrence Pilkington presided, a formal letter of resignation was read, and it was resolved to add Mr. Milner's name to the consulting staff. It was resolved also to convey to him the Board's high appreciation of the valuable services which he has always rendered during the period of his appointment. The Board sincerely wish for his rapid and complete recovery in his new home at Castletown, where they trust there are many happy years in store for him. Mr. Milner was elected honorary assistant surgeon to the hospital in October 1891, and was promoted to full surgeon in January 1898.

### A Woman Suffragist's Bequests to Hospitals.

Mrs. Rose Hyland, of Manchester, a member of the Manchester Board of Guardians, and of the militant section of the Women Suffrage Party, who left nearly £25,000, has made her will of special interest to hospitals on account of the bequests she has allocated to them and of the conditions under which she has made the bequests tenable. In each of the following cases Mrs. Hyland stipulated that the legacies shall not be payable unless within twelve months after her death the governing body of the beneficiary institution be made to include at least two women. She left £250 each to the Manchester Epileptic Colony, the "David Lewis" Consumptive Hospital (if still in existence), and to the Ancoats Hospital and Ardwick and Ancoats Dispensary; £150 each to the Hospital for Skin Diseases, Manchester, the Northern Counties Hospital for Incurables, St. Mary's Hospital, the Christie Hospital, Manchester, the Surgical Aid Society, Manchester, and the Ayr County Hospital; £130 to the Manchester Royal Eye Hospital; and £100 each to the Hospital for Consumption and Diseases of the Throat and Chest, Manchester, and to the Manchester Northern Hospital. Whatever opinions may be held on the questions which Mrs. Hyland had most at heart, everyone will admit the sense and zeal which made her will as active in encouraging them after her death, as she herself had been in her lifetime.

### Death of Dr. E. M. Grace.

It was of course in the world of cricket rather than in the hospital world that Dr. Edward Miles Grace was known, and his death has been a much-chronicled event in the sporting columns of the newspapers. The brother of "W. G.," qualified in 1865, having studied at Bristol and Edinburgh. His institutional appointments were few, but he had opportunities for useful professional work as Coroner for the Lower Division of Gloucestershire. He was also Surgeon to the Post Office and to the Oddfellows. The principal cricketing events of his life were his first match at Lords in 1861, his visit to Australia in 1863, and the thirty-one occasions on which in one innings he took all the wickets. In 1909 a testimonial for his services to Gloucestershire cricket and a cheque for £600 was handed to him by the Duke of Beaufort.

